# Structural Allele-Specific Patterns Adopted by Epitopes in the MHC-I Cleft and Reconstruction of MHC:peptide Complexes to Cross-Reactivity Assessment

**DOI:** 10.1371/journal.pone.0010353

**Published:** 2010-04-26

**Authors:** Dinler A. Antunes, Gustavo F. Vieira, Maurício M. Rigo, Samuel P. Cibulski, Marialva Sinigaglia, José A. B. Chies

**Affiliations:** Núcleo de Bioinformática do Laboratório de Imunogenética, Department of Genetics, Federal University of Rio Grande do Sul, Porto Alegre, Brasil; Albert Einstein Institute for Research and Education, Brazil

## Abstract

The immune system is engaged in a constant antigenic surveillance through the Major Histocompatibility Complex (MHC) class I antigen presentation pathway. This is an efficient mechanism for detection of intracellular infections, especially viral ones. In this work we describe conformational patterns shared by epitopes presented by a given MHC allele and use these features to develop a docking approach that simulates the peptide loading into the MHC cleft. Our strategy, to construct *in silico* MHC:peptide complexes, was successfully tested by reproducing four different crystal structures of MHC-I molecules available at the Protein Data Bank (PDB). An *in silico* study of cross-reactivity potential was also performed between the wild-type complex HLA-A2-NS31073 and nine MHC:peptide complexes presenting alanine exchange peptides. This indicates that structural similarities among the complexes can give us important clues about cross reactivity. The approach used in this work allows the selection of epitopes with potential to induce cross-reactive immune responses, providing useful tools for studies in autoimmunity and to the development of more comprehensive vaccines.

## Introduction

Cytotoxic T Lymphocytes (CTLs) recognize processed peptides presented in the context of the Major Histocompatibility Complex (MHC) class I, on the surface of nucleated cells [1]. The presented peptides, or epitopes, are short sequences with eight to twelve amino acids in length. These epitopes are derived from proteins endogenous to the cell and could derive from the cell itself or from intracellular parasites, making this pathway an important mechanism for antiviral defense [2]. The interaction between a T Cell Receptor (TCR) and the MHC:peptide complex (pMHC) is degenerated. It has been proposed that one single TCR can recognize up to 10^6^ different pMHCs [3]. This capacity of one TCR to recognize different epitopes defines the phenomenon of cross-reactivity [4].

The term “heterologous immunity” is used to describe a partial immunity induced against a pathogen through the host immunization with a non-related pathogen or antigen. This phenomenon, directly related to the mechanism of cross-reactivity, depends on the immunological history of the host and, consequently, on memory lymphocytes. Heterologous immunity can be involved in situations as diverse as pathogen clearance, chronic viral infection or autoimmunity induction [5]. There are several reports of heterologous immunity among non-related viruses and also reports on autoimmunity induced by molecular mimicry [6], [7], [8], but the mechanisms that establish such cross-reactions have not been completely solved [4]. Most studies on cross-reactivity are focused on very similar epitopes that share almost all amino acids [9]. However, cross-reactivity *in vitro* was already observed between epitopes that share less than 40% of their linear amino acid sequences [10]. Therefore, we need to look beyond the linear amino acid sequences to study or predict cross recognition between peptides. It is important to evaluate structural and chemical features such as amino acids physicochemical characteristics of the peptide [11], topology and electrostatic potential of the MHC:peptide complex [7], [8].

The HLA-A2-restricted NS3_1073_ epitope (CI/VNGVCWTV) is one of the main targets of the CTL response against Hepatitis C Virus (HCV) and has been also reported in cross-reactivity events [9], [12], [13]. In a work published in 2008, Fytili and colleagues investigated the relevance of each amino acid position of this epitope, for T cell recognition [13]. They tested alanine exchange peptides against NS3_1073_-specific CD8+ T cells and found that even single amino acid changes could almost completely abolish the production of interferon-ã by wild-type-specific T cells.

Molecular docking, as a bioinformatic tool, has been successfully used to both perform the complexation between a ligand and its receptor as well as to explore possible sites of interaction between a given compound and one protein of interest [14]. There have been several works describing the use of molecular docking in drug design, not only contributing to a better understanding of the functions of already described active compounds, but also adding to the development of new ones [15]. To refine the models generated by docking, an approach based on Energy Minimization (EM) can be used. This technique, normally performed in aqueous solution, induces the protein and its ligand to adopt a more stable conformation, closer to *in vivo* state [16], [17].

In the present work we performed a full search of the MHC structures available at the PDB, and identified conformational patterns shared by epitopes presented by a given allele. Besides an immunologically interesting finding, these features allow us to construct pMHC complexes with any peptide of interest. For instance, through the combined use of molecular docking and EM, we built the structure of the NS3_1073_ epitope in the context of the HLA-A*0201 allele (HLA-A2-NS3_1073_), aiming to identify the existence of molecular characteristics which may be involved in the stimulation of immune response. The strategy used to build this complex, followed by the use of softwares that enabled us to analyze structural and chemical features of the generated complexes, allowed us to study new pMHCs under the TCR “point of view”, and therefore, to infer a cross-reactive potential between two different epitopes [7], [8], [18]. In this context, peptide targets could be further synthesized for *in vitro* confirmation of their immunogenicity and cross-reactive potential against other targets of interest, such as immunodominant epitopes of related viruses. Once confirmed these features of interest, these targets could be used in polytope DNA vaccines, especially for heterologous prime-boost approaches [19], [20], [21], [22].

## Results and Discussion

### Identification of an allele specific structural epitope pattern

**Figure 1:**
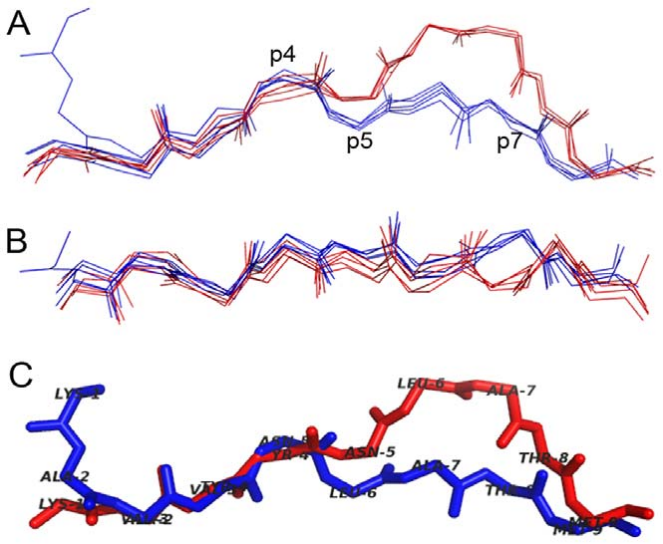
Superposition of H-2D^b^-H-2K^b^ -restricted epitopes. Structures of five H-2D^b^ -restricted epitopes (1CE6, 1S7V, 1WBZ, 1ZHB and 3BUY) and of five H-2K^b^-restricted epitopes (1FO0, 1FZJ, 1LK2, 1RJY and 1S7R) were superposed. The color pattern was kept on figures A, B and C. A: The side view shows the conformational differences among the H-2D^b^ (red) and the H-2K^b^ –restricted epitopes (blue), especially between positions 5 and 7 (p5-7). B: Top view of the 10 superposed peptides. C: Two crystal structures (1S7V and 1S7R) representing the same epitope (KAVYNLATM) in the context of these two different alleles.

**Figure 2:**
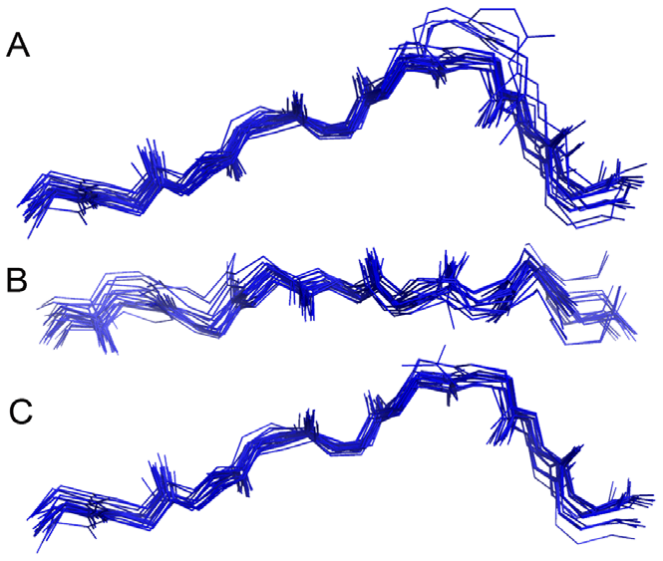
Conformational pattern of H-2D^b^-restricted epitopes. A: Crystal structures of 28 epitopes (Table 1) - size range between 8 and 11 amino acids - were superposed. It is possible to observe the existence of a shared pattern among the epitopes main chains (backbone). The orientation (N-terminal to C-terminal) was kept. B: Top view of the same 28 epitopes. C: Only the epitopes with nine amino acids were superposed, showing that the variability among the epitopes structure is determined by differences in length. The side chains of the epitopes were not represented in these images, and an amino acid (p7) of epitope 2VE6 (altered photocleavable peptide) was excluded.

We have performed a search for all MHC-I structures deposited at PDB and have analyzed the conformation of different epitopes presented by these MHC molecules. A conformational pattern, specific for each allele, has been observed, evidencing that the tridimensional structure of the presented epitope is not solely an intrinsic characteristic of the epitope itself (i.e. due to the amino acid sequence *per se*), but is actively determined by the MHC-I cleft, which varies according to the MHC allele (Figures 1 and 2). The structural data of the epitopes capable to bind to the H-2D^b^ and H-2K^b^ molecules (the murine MHC alleles with the highest number of structures at the PDB) were analyzed taking into consideration both their anchor positions as well as the cleft structure. The results indicated the regions of the epitope that suffer a restraint and those that could accept variations in the amino acid side chains. We postulate that more restrained regions were related to the MHC binding although regions more “flexible” and exposed would contact with the TCR, therefore defining the pMHC specificity (Figure S1).

Although the sequence of some of the epitopes included in this analysis differs by just one amino acid, what could bias the identification of a general pattern of an allele, epitopes with far different sequences maintained the same pattern. Moreover, variants of the same epitopes were included based on the idea that the exchange of any amino acid can induce an alteration in the backbone torsions and even abrogate the recognition by a TCR [13], [23]. When analyzing the structures, we verified that exceptions to the pattern occur as a requirement needed to accommodate longer sequences on the MHC cleft. We also realized that structural patterns are specific for a given MHC allele, so that epitopes presented by different alleles shows different conformations (Figure 1A). Furthermore, previous works already indicated that a same epitope will adopt different conformations when presented by different MHC alleles (Figure 1C) [24]. Together, these data indicate that the epitope conformation in the MHC cleft is determined both by its linear sequence and the cleft topology.

**Figure 3:**
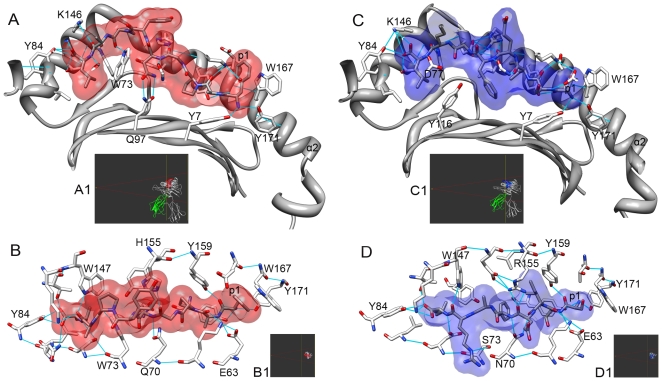
Interactions between epitope and MHC cleft. A: Images of H-2D^b^ (1CE6) and H-2K^b^ (1RJY) complexes are generated with Chimera package. Position of the complexes is indicated in the Side View Window (A1,B1,C1,D1). H-2D^b^-restricted peptide is depicted in red (A, B) and H-2K^b^-restricted peptide is depicted in blue (C,D). MHC side chains that interact with the peptide are represented as sticks and Hydrogen bonds are represented as light blue lines. The MHC Alpha-1 domain (α1) and the first position of the peptide (p1) are indicated, as also some amino acid labels.

During the peptide loading process, the epitope is induced to adopt an adequate conformation and to do so, some features specific for each MHC allele are required. Most of these features had already been discussed in literature, but they have not as yet been directly related to conformational patterns of the presented epitopes. For instance, the differences between peptides restricted to H2-D^b^ and H2-K^b^ can be partially explained based on the pattern of hydrogen bounds generated between the peptide and the MHC molecule in each allele [25] and the presence of a tryptophan residue (Trp73), in the D^b^ molecule [26] (Figure 3). The Alpha-2 domain (α2) of MHC-I molecules is constituted by two segments of α-helix joined by a little loop that protrudes from the cleft. We verified that in most cases the peptide backbone follows the α2 structure, having a bulge at the C-terminal, in the same region of the bulged coil. Part of this bulge is probably induced by the presence of a tryptophan residue (Trp147) in the α2 domain that is highly conserved in several MHC alleles. H2-D^b^-restricted epitopes have an even higher bulge at C term (p6-8) because this allele has a second tryptophan residue (Trp73), in the same region, but from the α1 domain. These two residues almost close the cleft, forcing the peptide to pass above them (Figure S2). An alignment of all entire sequences of H2-D^b^, H2-K^b^ and HLA-A*0201 available at PDB, has revealed the presence of the Trp147 in all sequences of these alleles. All the sequences of H2-D^b^ also have the Trp73 residue. The H2-K^b^ sequences have serine in position 73 of α chain. The absence of the tryptophan results in a deeper cleft in this region. Besides, we verified that in some cases (e.g. 1LK2) the serine residue at this site also establishes an hydrogen bound with the epitope at p5, that is one of the anchor positions of the H2-K^b^ epitopes (data not shown). In the HLA-A*0201 allele, the residue at position 73 is a threonine (Thr73) and does not seem to be involved in any hydrogen bond with the epitope. However, this allele has another highly conserved residue (Asp77) that establishes H bonds with the epitope C-terminal.

A main conformation for the backbone residues of the 9-mer peptides restricted to the human allele HLA-A*0201 was also identified in our work (Figure S3), although when compared to murine described patterns, a greater divergence was observed. Interestingly, most of the divergent peptides are cancer related and, as already discussed in the literature, cancer related peptides present great conformational variation [27]. Deviation of the allele-specific pattern may not prevent the presentation on this allele, but might interfere either in the stability of the complex or in CTL stimulation. According to that, there is at least one example of deviation from the pattern and reduction of TCR recognition. Buslepp and colleagues published a paper on the crystal structures of three HLA-A2:peptide complexes [28]. One of them was an agonist (1I7U) of the TCR and the others were described as null (1I7T) and antagonist peptides (1I7R). These structures were herein superposed to other 31 HLA-A*0201-restricted peptides and the agonist fit exactly with the described pattern, while the other two peptides deviate from this pattern (Figure S3).

We also looked for these conformational patterns in B*0801, B*2705 and B*35 (Figure S3 and Table S1). We believe that the existence of patterns could be extrapolated to other alleles, even those of other species. However, this cannot be demonstrated now, since the number of non-human/non-murine MHC-I structures available is a very small.

Although the structural pattern observed applies only to the backbone of the peptide, this does not imply a random distribution of the side chains along the cleft. In fact, a more detailed study of the 28 H2-D^b^ ligands (Table 1) evidenced the existence of regions with different degrees of restriction over the amino acids side chains. Epitopes in the H2-D^b^ cleft are positioned in such a way that a portion of its amino terminal (N-terminal) end is hidden under the MHC side chains (p2–3 of the epitope) while another portion (p6–7) is out of the MHC cleft (Figure S1A). Not surprisingly, there is higher identity among side chains of different epitopes in p2–3 and higher diversity in p6–7 (Figure S1B). The p6–7 region, where the side chains are more diverse among different epitopes, will contact the TCR and therefore will determine the TCR specificity. We also observed low diversity among side chains in positions p5 and p9, described as the anchor positions of this allele. An alignment of the 28 H-2D^b^ ligand sequences, considering its physicochemical characteristics, indicated a preferential occurrence of an asparagine on the fifth position, although in three cases (1INQ, 1JUF and 1BZ9) a nonpolar amino acid occupied this position. Most of the H-2D^b^ ligands had nine amino acids; however, we also found 10-mers and 11-mers. The last position (p9, p10 or p11) was also conserved. Almost all sequences presented nonpolar amino acids at this site, except for one epitope, which had a cysteine (1FG2). Despite the differences in length, the anchor positions (p5 and the last amino acid) were conserved and length adjustments occurred at p6–7, with foldings outside the cleft. At this region a higher divergence could be observed, even in the backbone itself (Figures 2A and 2C). One of the ligands, the synthetic peptide FAPGVFPYM (1BZ9), highly deviates from the backbone pattern, especially at p5, rising up out of the cleft (Figure S4). This epitope does not possess the canonical anchor residue at p5 and, as briefly discussed in the original reference, peptides may occasionally bind without primary anchor residues if other residues within the peptide compensate the lost binding energy [29]. This could account for its unusual shape, although it is important to stress the fact that this is a synthetic peptide.

**Table 1:** List of H-2D^b^ ligands available at PDB.

PDB code	Epitope description	Sequence (aa)	N° of aa
1FG2*	LCMV Peptidic Epitope (gp33)	LAVYNFATC	9
1JPF*	LCMV Peptidic Epitope (gp276)	SGVENPGGYCL	11
1JPG*	LCMV Peptidic Epitope (np396)	FQPQNGQFI	9
1INQ*	MHC Class I H13^a^ minor histocompatibility peptide	SSVVGVWYL	9
1JUF*	Minor Histocompatibility Antigen peptide (H13^b^) P4(Val/Ile)	SSVIGVWYL	9
1N3N*	Mycobacterial hsp60 epitope	SALQNAASIA	10
1QLF*	Sendai NP_324-332_ (*gly327 replaced by o-glcnac serine*)	FAPSNYPAL	9
1S7U/2F74/ 1FFN/1N5A*	LCMV-derived immunodominant peptide (gp33)	KAVYNFATM	9
1S7V*	LCMV-derived (gp33) index peptide - escape variants	KAVYNLATM	9
1S7W*	LCMV-derived (gp33) index peptide - escape variants	KALYNFATM	9
1S7X*	LCMV-derived (gp33) index peptide - escape variants	KAVFNFATM	9
1FFO*	Gp33 syntethic peptide with substitution K1A /C9M	AAVYNFATM	9
1FFP*	Gp33 syntethic peptide with substitution K1S /C9M	SAVYNFATM	9
1WBX*	Peptide from Influenza A (pr8) HA_468-477_	SQLKNNAKEI	10
1WBY/1YN6*	Peptide from Influenza A (pr8) PA_224-233_	SSLENFRAYV	10
1YN7*	Mutated peptide (R7A) from RNA polymerase subunit P2	SSLENFAAYV	10
1ZHB*	9-mer peptide from Dopamine beta-monooxygenase	KALYNYAPI	9
1BZ9*	Synthetic Peptide (P1027)	FAPGVFPYM	9
1CE6*	Sendai Virus Nucleoprotein Peptide (NP_324-332_)	FAPGNYPAL	9
2VE6*	Altered peptide of Sendai virus (*Photocleavable peptide*)	FAPGNY**P**AL	9
2CII	Sendai virus nucleoprotein epitope	FAPGNYPAL	9
2ZOK*	9-meric peptide from Spike glycoprotein	**A**SLWNGPHL	9
2ZOL*	9-meric peptide from Spike glycoprotein - Mutation: W4S	**A**SLSNGPHL	9
1HOC*	Influenza virus peptide NP_366-374_	ASNENMETM	9
3CPL*	Influenza virus peptide NP_366-374_ with substitution M6A	ASNENAETM	9
3BUY*	Epitope of PB1-F2	LSLRNPILV	9
3CC5*	Nonameric peptide from Melanocyte protein Pmel 17	KVPRNQDWL	9
3CCH*	nonameric peptide murine gp100	EGSRNQDWL	9
3CH1*	nonameric peptide chimeric gp100	EGPRNQDWL	9

Thirty-three files containing H-2D^b^ alleles were found in PDB, 28 (*) presented different epitopes. These 28 epitopes were used at structure and sequence analyses. 1FFO presents an MHC interacting with the TCR. Since this interaction could affect the conformation of the epitope, this structure was excluded. Structures presenting incomplete epitope sequence have been also excluded (e.g. 2CII). 1BZ9 epitope was included in all analyses, however it was excluded in Figure 1 and Figure 1SB.

The H-2K^b^ ligands analyses also demonstrated a conservation of the side chains in amino acids presented at anchor positions. Of the 21 analyzed sequences (Table S2), 16 have aromatic amino acids (F or Y) at p5, while four of the remaining sequences presented polar amino acids (N, S or T) at this position. The epitope from 1WBZ was the only one with a positively charged amino acid at p5, but this epitope presents a non-canonical binding motif which may have some influence over the immunodominance [26]. All sequences had a nonpolar amino acid at the last position (C-term). In comparison to the H-2D^b^ allele, H-2K^b^ seems to be less restrictive to peptide ligands, allowing a greater divergence even at p4 (Figure S5).

### Establishment of a strategy to build pMHC compIexes

Considering that the backbone structure of the epitope is shaped by its direct interaction with the MHC cleft, we can postulate that epitopes capable to be presented by a given MHC allele will adopt rather similar conformational structures to other peptides presented by the same allele. This has been the premise for developing a new approach that allows us to build pMHCs whose crystal structures were unavailable. In this approach, we use a set of epitopes — already determined by crystallography in the cleft of the MHC-I of interest — to choose a standard epitope structure. Thereafter, this standard is being used as a template to build the structure of another epitope (see methods).

This strategy has been used to reproduce the crystal structure of four different pMHCs available at PDB (Table 2). For instance, we reproduced the HLA-A2-P17 complex, PDB access number 2V2W, using as template a different HLA-A2-restricted epitope (SL9-1A/6A/8A)[13]. The structures have been fit by MHC C^α^ atoms and a Root Mean Square Deviation (RMSD) value of 0.96 Å (for all epitope atoms) was obtained. This value accounts not only for the torsional differences between the reproduced epitope and the crystal, but also for differences in epitope position inside the cleft. According to the literature, RMSDs lower than 2.2 Å are considered valid reproductions [30], [31]. In order to assure the reproducibility of the used approach, we also reproduced the entire set of available class I PDB structures for the alleles with established peptide pattern. We were able to reproduce a total of 46 structures —11 HLA-A*0201, 19 H2-D^b^ and H2-K^b^ — with RMSD values of 1.754±0.4675 Å (all peptide atoms), which characterizes a high fidelity reconstruction index. Exceptions to the predicted pattern, as previously discussed, were not reproduced.

**Table 2:** Reproduction of pMHC crystal structures.

Allele	Ep. Length	Target	MHC donor	Pattern Template	BE D1^a^ (Kcal/mol)	BE D2^b^ (Kcal/mol)	RMSD^c^	RMSD^d^
H2-D^b^	9	1HOC	1WBX	1JPG	−11,2	−12	1,22	1,48
H2-D^b^	10	1WBX	1WBX	1WBY	−11,2	−12,3	1,49	1,53
H2-K^b^	8	1LK2	1LK2	1RJY	−11,8	−13,6	1,28	1,45
HLA-A*0201	9	2V2W	2V2W	1T1Z	−12,3	−12,9	0,89	0,96

Four different pMHC crystal structures available at PDB were reproduced. A docking approach based in the allele-specific patterns was performed (see methods). Information about the MHC alleles, the PDB files and the results are presented in this table.

a Binding Energy of the first docking.

b Binding Energy of the second docking.

c Root Mean Square Deviation for all epitope atoms. Calculated after fit the epitopes by C alpha.

d Root Mean Square Deviation for all epitope atoms. Calculated after fit the MHCs by C alpha.

Considering that the proposed approach successfully reproduced a large set of structures from three different MHC-I alleles, and also reproduced the structure of epitopes with different lengths inside the cleft of the same allele (H2-D^b^), we can use this computational strategy to built pMHC complexes based on the linear amino acids sequence of any epitope. It should be noted that not all peptides can be presented by a given MHC allele. We believe that the docking process can identify “bad ligands” through the variation of the binding energy. However, this approach becomes more reliable if preceded by an analyses of epitope prediction or, at least, by the verification of the affinity between the epitope and the MHC of interest, through the use of a MHC ligands databank.

Several works focused on MHC Class-I binding peptide prediction [32], [33], [34], [35], [36], [37] and it is important to emphasize that this is not the aim of our approach. The allele-specific patterns and the combined use of Docking and Energy Minimization (*D1-EM-D2*) are presented here as a tool to construct new pMHC complexes, which can be further analyzed using several available programs. There are few alternative methodologies to construct pMHC complexes, and they present several limitations regarding the MHC alleles or the epitope length [37], [38], [39], [40]. Although it has its own limitations, our approach can be applied to different epitope lengths and MHC alleles, being also more reliable, as it uses an established conformational pattern for each allele as template for the peptide. Therefore, the realistic prediction of pMHC complexes remains an important goal in peptide vaccine design and here we describe a new approach that will certainly contribute to this field.

### *In silico* study of cross-reactivity potential between the wild-type complex HLA-A2-NS3_1073_ and nine pMHCs presenting one amino acid changed by alanine

In order to analyze cross reactive potential, Fytili et al. 2008 [13] tested *in vitro* the T cell stimulation capacity of an HCV wild-type epitope NS3_1073_ (CINGVCWTV) and nine other epitopes derived from this original, each one being an alanine exchange variant, against a cell population previously exposed to the original epitope. In our work, the presently described docking approach was used to built the pMHC complexes of the HCV wild-type epitope NS3_1073_ (CINGVCWTV) and the nine other epitopes in the context of the human allele HLA-A*0201, aiming at analyzing structural and chemical features of these complexes. The accessible surface area (ASA) of the peptides in the cleft was also calculated, and interesting results came out. The plot of these values for each residue has presented quite identical signatures among the peptides that induced strong response *in vitro* (Figure S6). Some works have associated high ASA values and peptide immunodominance [26]. This relation was not seen in our study, whereas some epitopes with poor response, *in vitro,* presented high values of ASA, especially at p5. However, it is important to note that the peptide with the lowest value of ASA at p5 (Seq3) was the only peptide that does not stimulate a detectable response [13]. In order to provide further evidence for the importance of the ASA value, we also performed the construction and analysis of 28 pMHC complexes, from 6 different HCV genotypes, described by Fytili et al. 2008. In their description, the experimental data showed a cross-genotype-reactivity, particularly between the wild type peptide (Peptide 1 from Genotype 1 or G1_1) and the peptides from genotype 6 (G6_23 to G6_28). In agreement with this data, the ASA values from genotype 6 presented the same pattern of the wild type peptide (Figure S7) and the peptides with higher deviation from this “ASA pattern” (G3_16, G3_18 and G3_20), presented the lowest levels of IFN-γ production in all ELISPOT assays (Fytili et al. 2008). However, it is also important to note that some peptides with low response in vitro presented ASA values quite similar to the wild type. These results support the idea that large deviations in ASA values may indicate a poor stimulation in vitro, but the opposite is not true. Therefore, the ASA values are just an indicator that may be taken into account during a screening for possible targets, and cannot alone explain all variation observed *in vitro*.

**Figure 4:**
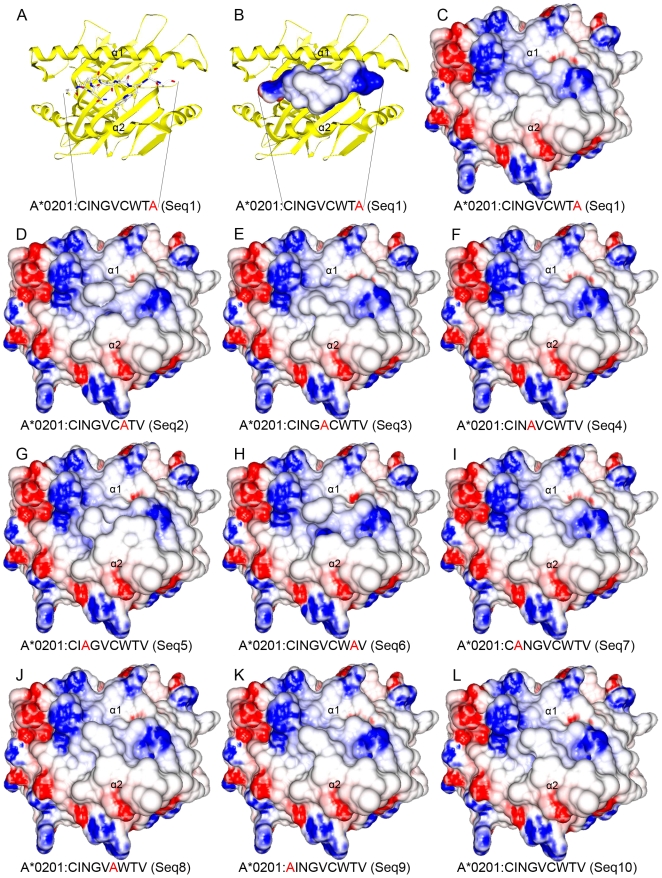
Topology and electrostatic potential comparison among A2*0201:HCV complexes. TCR-interacting surfaces of ten different pMHC complexes are depicted (C–L). Regions with positive (blue) and negative (red) charges are represented with a scale from −10 to +10 kiloteslas. Sequence of presented peptide is depicted above each complex and the position of the Alanine exchange is depicted in red. Alpha-1 and Alpha-2 MHC domains are also indicated. Images A, B and C show the same pMHC, in the same position, size and orientation, although with different representations. TCR-interacting surfaces of complexes that stimulate high levels of IFN-γ production by CVNGVCWTV-specific CD8+ T-cells (J,K and L) shared both topology and electrostatic potential. One complex that stimulates low levels of IFN-γ production (G) presented differences in topology and reduction of a negatively charged region in the TCR-interacting surface.

A previous work have already determined the importance of the epitope central amino acids for the T cell recognition [5]. As described by Fytili et al. 2008 [13], alanine exchanges at p3, p4, p5 and p7 strongly affected T cell stimulation capacity. However, the peptide with the C6A exchange stimulated T cells at similar levels as compared to the wild-type epitope. This is an important change in terms of physicochemical characteristics and is quite curious that this does not seem to affect T cell recognition. We have analyzed the TCR-interacting surfaces of all complexes concerning their topology and electrostatic potentials. The results indicated that this C6A substitution has almost no effect in the pMHC interacting surface (Figure 4J). In agreement with the similar ASA values, the complexes 8, 9 and 10 presented quite identical topologies and charge distribution. We therefore postulate that this similarity is the main aspect responsible for the described cross recognition of these complexes by the CVNGVCWTV-specific CD8+ T cells [13].

Using the same dataset, the pMHC surfaces can also provide information about the peptides that poorly stimulate the production of interferon-ã. For instance, the peptide number 5 (CIAGVCWTV) was the only out of ten peptides, in which the tryptophan indole ring had turned to the C terminus end of the peptide. This change of position caused not only a difference of topology but also a reduction of a positively charged area at the pMHC surface (Figure 4G). To ascertain that this observation was not spurious, the steps to construct this complex were repeated using a higher value of exhaustiveness ( = 15) at AutoDock Vina program [31]. As discussed before, complex 3 presented the lowest ASA value at p5, and this value reflects the existence of a less bulging side chain at this site (Figure 4E). Analysis of the pMHCs surface can help us predict cross reactivity potential, especially when the complexes are very similar. However, to explain all the variation of an immune response in terms of topology and potential is certainly impossible. Complex 2 also has differences in topology as compared to complex 10, but it is structurally similar to complex 6, which stimulates an intermediated response. Differences in other points of the antigen processing and presentation pathway (or other related pathways) will probably be responsible for such different capacities to induce an immune response. For instance, we could suggest that a different stability of those complexes could be responsible for a reduction, both in number or in time of exposure, of those complexes on the presenting cells surface. This would reduce the probability to establish TCR:pMHC contacts long enough to activate the CTLs.

In conclusion, our work provides a new approach to build up and *in silico* analyze pMHCs. The structural allele-specific patterns identified can be incorporated in other immunoinformatic approaches, in order to provide more information about peptide affinity for specific MHC-I alleles and the immunogenicity of the resulting complexes. The new approach to construct pMHC-I complexes helped us understand some of the differences in the CD8+ CVNGVCWTV-specific response and could be applied to other studies of cross-reactivity potential among any epitopes of interest. As previously mentioned, these peptide targets could be further synthesized for in vitro confirmation of their immunogenicity and cross-reactive potential against other targets of interest, such as immunodominant epitopes of related viruses. Once these features of interest are confirmed, these targets could be used in polytope DNA vaccines, especially for heterologous prime-boost approaches [20], [21], [22].

## Methods

### Sequence alignment

The sequences were analyzed using the Jalview multiple alignment editor [41]. All sequences of each MHC allele were separately aligned with the muscle algorithm. Epitope sequences were aligned according to its physicochemical characteristics using the software Bioedit 7.0.5.3. [42].

### Construction of pMHC complexes

The pMHC complexes construction was performed through the steps presented on Figure S8. First, we identified a PDB structure (e.g. complex “A”), wich includes the MHC allele of interest and an epitope (template epitope “a”) with the same number of amino acids of the epitope that we want to model (e.g. “c” epitope). Then, the structures of the MHC and the epitope (from “A” complex) were separated, saving the epitope structure in an independent file that will be opened with the SPDBV 3.7 [43]. Using the *Homology modelling* menu of this software (SwissModel), with *Magic Fit*, the FASTA sequence of the epitope to be modelled (“c” epitope) is superposed to the structure of the template epitope “a” (acquired from “A” complex). The “fit” structure of “c” epitope may have some atoms in energetically unfavorable positions or amino acids with unfit torsions. These were corrected running a short EM (nsteps  = 100) with the GROMACS 3.3.3 package [44]. At this point, we have a 3D structure related to our epitope of interest for the first time. This structure was subjected to a first docking (*Dock1*) with a MHC molecule. In this step the MHC molecule can be those from the “A” complex or from another PDB file with the same MHC allele of interest. The epitope originally presented by this “MHC donor” structure should not interfere in the analysis. It is quite important to use a PDB file with the best possible resolution. All MHC bonds are maintained rigid during the docking, as most of the epitopes backbone bonds, since the epitopes main chain is already in the suitable conformation. During the docking procedure, the epitope side chains are flexible, which allows a great conformational variation. At this point, a 3D model of the pMHC complex is available, but more steps are needed to refine this model. The resulting pMHC, with the best docking result (or the most frequent) is subjected to a longer EM (nsteps  = 1000). This step is important to adjust the MHC side chains that interact with the new ligand, reducing unfavorable interactions. The last step is the separation of the two components (MHC and epitope) from this minimized complex and the use of both structures as inputs to a second docking (*Dock2*) that will generate the pMHC of interest. The second docking allows the docking program to search all over again for the best epitope conformation. The influence of pMHC minimization over the docking results is confirmed by the improvement of binding energies (see Table 2).

### Molecular Docking

The molecular dockings were performed with AutoDock Vina 1.0.2 [31] using default values (e.g. exhaustiveness  = 8). For each epitope, the same input file was used to run AutoDock Vina 20 times, generating a final population with a thousand different conformations (20 outputs with 50 structures each). The best conformation of each output was open together in PyMol viewer and was analyzed according to the frequency of occurrence and binding energy values. One of the most frequent conformations was selected to represent the final conformation of the epitope in the MHC-I cleft. The running time of a simulation will vary accordingly to the computational power used, and the number of rotatable bonds of the epitope of interest. Using our default configuration, the running time of each docking step (20 rounds) is about three hours in a quad core computer (using the four cores at full capacity) and the whole process to get a pMHC complex (*D1-EM-D2*) takes about 7 hours in the same scenario.

### Accessible Surface Area (ASA) analyze

The ASA values were calculated in the NOC 3.01 program [45]. The obtained values were plotted at the Microsoft Office Excel 2007 software.

### Construction of the HLA-A2-NS3_1073_ complex and the nine variants

The PDB structure 2V2W was used as “MHC donor” and the C chain of 1T1Z structure was used as template for the HLA-A*0201 epitopes pattern. The ten complexes were independently constructed and analyzed as previously described (Construction of pMHC complexes).

### Analyses of topology and charges distribution

The MHC surface analyses were performed with the GRASP2 program [46], on Windows XP platform. The electrostatic potential was calculated with a scale from −10 to +10 kiloteslas.

### Images acquisition

The epitopes superposition was performed using SPDBV 3.7 [43], though the images were acquired with PyMOL 1.0 program [47]. The pMHC top view images were generated with the GRASP2 program [46]. Images of MHC:peptide interactions were produced using the UCSF Chimera package from the Resource for Biocomputing, Visualization, and Informatics at the University of California, San Francisco (supported by NIH P41 RR-01081)[48]. All images were edited with Adobe Photoshop CS2 v.9.0. program.

## Supporting Information

Table S1 List of ligands used to identify HLA-restricted patterns.
(0.09 MB DOC)

Table S2 List of H-2K^b^ ligands available at PDB.
(0.04 MB DOC)

Figure S1 Structural organization of the H-2D^b^-restricted epitopes. A: Images of HBsAg_30-39_ epitope (presented in *Ball and Stick_CPK*) in the cleft of the H-2D^b^-allele (represented as surface, with negatively (red) and positively (blue) charged regions with a scale from −10 to +10 kiloteslas). Partial N-terminal ending of the epitope is hidden under MHC side chains (p2-3). Some regions of the peptides (p6-7) protract out to the MHC cleft. B. Superposition of 28 structures of H-2D^b^-restricted epitopes (Table 1), including side chains. It is possible to observe a higher variability in the protracted region as compared to the N-terminal and to the anchor sites (p5 and p9). The position of the side chains of amino acids 1, 5, 7 and 9 are shown in both images.
(3.57 MB TIF)

Figure S2 Topology of H2-D^b^ and H2-K^b^ binding clefts. A: Crystal structure of an H-2D^b^ allele (PDB access code 1CE6) is depicted as Ribbon and Surface. Epitopes inside the cleft are depicted as Sticks. Two tryptophanes of the MHC alpha-chain (W73 and W147) almost block the cleft, forcing the peptide to pass above them. B: Crystal structure of an H-2K^b^ allele (PDB access code 1RJY) is depicted with the same configuration. The absence of tryptophan (W73) results in a deeper cleft in this allele.
(4.42 MB TIF)

Figure S3 Conformational patterns of human MHC alleles. A: Thirty-four HLA-A*0201-restricted peptides (Table S1) were superposed using SPDBv program. Peptides sharing an A*0201-restricted pattern are depicted in blue. Exceptions to this pattern are depicted in cyan. Cancer-related peptides are depicted in light pink. Epitopes 1I7R and 1I7T are depicted in red (see Discussion). B: Superposition of five B*0801-restricted peptides. C: Superposition of five B*2705 restricted peptides. D: Superposition of three B*3501-restricted and two B*3508-restricted peptides. In this case, we have not enough structures to predict a pattern. Besides that, we can see that both B*3501-restricted 9-mers (blue) presented a similar conformation. In addition, a 10-mer peptide (APQPAPENAY) presented almost the same conformation when presented by B*3501 (orange) and B*3508 (red). A B*3508-restricted 8-mer is also depicted (green).
(3.56 MB TIF)

Figure S4 Exception to the H-2D^b^ pattern. A: The 1BZ9 epitope (red) does not have the conventional amino acid in the anchor position (p5) and showed a significant deviation in the main chain when compared to other epitopes. B: The side chains of the epitope 1BZ9 (red) are in a similar conformation to other epitopes of this allele, except in a phenylalanine at position 6 of the epitope, which may be used as an alternative “anchor”. Observe the presence of an anchor amino acid in C-terminal and a hidden N-terminal extremity under the side chains of the MHC, characteristics that may contribute to the presentation of this unusual epitope.
(2.86 MB TIF)

Figure S5 Structural pattern of H-2K^b^ restricted ligands. A: Twenty-two epitopes (see Table S2) restricted to this allele were superposed, four 9-mer (red) and 18 8-mer (blue). The H-2K^b^ restricted ligands, just like those restricted to the H-2D^b^ allele, presented a higher identity in the side chains of the anchor positions than in the side chains oriented outside of the cleft. B: Backbone superposition of the 21 ligands shows a shared conformation among the epitopes with the same length (8-mer). The length adjustment, in this allele, seems to be in different positions, when compared to the H-2D^b^ allele.
(2.86 MB TIF)

Figure S6 Analyze of HCV alanine exchanged peptides. The wild type HCV derived peptide (CINGVCWTV) and nine alanine exchanged peptides were analyzed. A: Sequences of 10 peptides are indicated. Level of IFN-Î^3^ production by CVNGVCWTV-specific CD8+ T-cells, induced by each sequence, are also represented [13]. Each bar (in black) represents the number of the Spot Forming Units (SFU/10^4^ cells) produced by each of the peptides. Accessible Surface Area (ASA) plot of sequences that stimulates low (B), intermediated (C) and high (D) IFN-γ production are depicted. ASA values are measured in square angstroms. The wild type sequence (S10) was included in all plots.
(6.62 MB TIF)

Figure S7 Flowchart of a new pMHC complex construction. ASA values of the wild type HCV derived peptide (CVNGVCWTV) and 28 naturally occurring NS3_1073_-variants were analyzed. In agreement with experimental data, the ASA values from genotype 6 presented the same pattern of the wild type peptide and the peptides with higher deviation from this “ASA pattern” (G3_16, G3_18 and G3_20), presented the lowest levels of IFN-γ production in all ELISPOT assays (Fytili et al. 2008).
(2.15 MB TIF)

Figure S8 Flowchart of a new pMHC complex construction. Consider an epitope “C” whose structure in the context of a given MHC allele was not determined. A search at PDB is performed, looking for a PDB file containing the allele of interest presenting an epitope with the same length of the “c” epitope. In this example, we found the “A” complex. Using the SPDBV program, it was observed that the amino acid sequence of the epitope “c” is “*Fit*” on the 3D structure of the epitope present at the “A” complex. The generated structure of the epitope “c” is submitted to an energy minimization (EM), and is used as input for the docking with an “MHC donor” structure (B). In order to adjust the MHC to this new epitope, an EM of the complex “D” is performed. After minimization, epitope and MHC are separated and used as inputs to a second docking, which will generate the desired pMHC complex (E). For more information see methods.
(2.86 MB TIF)
