# Supplementary material for: Structural Allele-Specific Patterns Adopted by Epitopes in the MHC-I Cleft and Reconstruction of MHC:peptide Complexes to Cross-Reactivity Assessment
Source: PLoS One. 2010 Apr 26;5(4):e10353. doi: 10.1371/journal.pone.0010353 (PMC2860844; doi:10.1371/journal.pone.0010353)
Supplement: Table S1 — List of ligands used to identify HLA-restricted patterns. (0.09 MB DOC) [file pone.0010353.s001.doc]

### Table S1 – List of ligands used to identify HLA-restricted pstterns.

| **Allele** | **PDB code** | **Epitope description** | **Sequence (aa)** | **N° of aa** |
| --- | --- | --- | --- | --- |
| A2*0201 | 1JHT | Peptide Ligand From The MART-1/Melan-A   Mutation: A28L | ALGIGILTV | 9 |
| A2*0201 | 1S9X | NY-ESO-1 Peptide Analogue S9A | SLLMWITQA | 9 |
| A2*0201 | 1S9Y | NY-ESO-1 Peptide Analogue S9S | SLLMWITQS | 9 |
| A2*0201 | 2VLL | Flu Matrix Peptide | GILGFVFTL | 9 |
| A2*0201 | 1I7R | Peptide P1058 | FAPGFFPYL | 9 |
| A2*0201 | 1I7T | Peptide P1049-5v | ALWGVFPVL | 9 |
| A2*0201 | 1I7U | Peptide P1049-6v | ALWGFVPVL | 9 |
| A2*0201 | 2V2W | Hiv P17 | SLYNTVATL | 9 |
| A2*0201 | 2V2X | Hiv P17 | SLFNTVATL | 9 |
| A2*0201 | 1S8D | HIV Gag Peptide | SLANTVATL | 9 |
| A2*0201 | 1T1W | Hiv Gag Peptide | SLFNTIAVL | 9 |
| A2*0201 | 1T1X | Hiv Gag Peptide | SLYLTVATL | 9 |
| A2*0201 | 1T1Y | Hiv Gag Peptide | SLYNVVATL | 9 |
| A2*0201 | 1T1Z | Hiv Gag Peptide | ALYNTAAAL | 9 |
| A2*0201 | 1T20 | Hiv Gag Peptide | SLYNTIATL | 9 |
| A2*0201 | 1T21 | Hiv Gag Peptide | SLYNTVATL | 9 |
| A2*0201 | 1B0G | Peptide P1049 | ALWGFFPVL | 9 |
| A2*0201 | 1DUZ | Htlv-1 Octameric Tax Peptide | LLFGYPVYV | 9 |
| A2*0201 | 1EEY | Gp2 Peptide; Mutation: I2l,V5l,L9v | ILSALVGIV | 9 |
| A2*0201 | 1EEZ | Gp2 Peptide | ILSALVGIL | 9 |
| A2*0201 | 1HHG | Hiv-1 Gp120 Envelope Protein (Residues 195-207) | TLTSCNTSV | 9 |
| A2*0201 | 1HHJ | Hiv-1 Reverse Transcriptase (Residues 309-317 | ILKEPVHGV | 9 |
| A2*0201 | 1TVB | Epitope Of Melanocyte Protein Pmel 17 | ITDQVPFSV | 9 |
| A2*0201 | 1TVH | Epitope Of Melanocyte Protein Pmel 17 | IMDQVPFSV | 9 |
| A2*0201 | 2GIT | HTLV-1 TAX Peptide | LLFGKPVYV | 9 |
| A2*0201 | 1QEW | Protein (Melanoma-Associated Antigen 3) | FLWGPRALV | 9 |
| A2*0201 | 1I1F | Protein (Hiv-Rt Variant Peptide I1f (Flkepvhgv) | FLKEPVHGV | 9 |
| A2*0201 | 1I1Y | Hiv-Rt Variant Peptide I1y (Ylkepvhgv | YLKEPVHGV | 9 |
| A2*0201 | 1QR1 | Gp2 Peptide | IISAVVGIL | 9 |
| A2*0201 | 2GTW | Octapeptide From Melan-A/MART-1 | LAGIGILTV | 9 |
| A2*0201 | 2GTZ | Octapeptide From Melan-A/MART-1; Mutation: A28L | ALGIGILTV | 9 |
| A2*0201 | 2GUO | Native nonameric Melan-A/MART-1(27-35) peptide | AAGIGILTV | 9 |
| A2*0201 | 3FQT | Peptide 38-46 From Cell Division Cycle 25b (CDC25b) | GLLGSPVRA | 9 |
| A2*0201 | 3FQW | Peptide 1097-1105 From Insulin Receptor Substrate 2 (IRS2) | RVASPTSGV | 9 |
| B*0801 | 1AGB | Hiv-1 Gag Peptide (3r Mutation) | GGRKKYKL | 8 |
| B*0801 | 1AGC | Hiv-1 Gag Peptide (7q Mutation) | GGKKKYQL | 8 |
| B*0801 | 1AGD | Hiv-1 Gag Peptide (Index Peptide) | GGKKKYKL | 8 |
| B*0801 | 1AGE | Hiv-1 Gag Peptide (7r Mutation) | GGKKKYRL | 8 |
| B*0801 | 1AGF | Hiv-1 Gag Peptide (5r Mutation) | GGKKRYKL | 8 |
| B*2705 | 1JGE | Peptide M9 | GRFAAAIAK | 9 |
| B*2705 | 1W0V | Self-Peptide Tis From Egf-Response Factor 1 | RRLPIFSRL | 9 |
| B*2705 | 2BSR | Epstein-Barr Nuclear Antigen-6 | RRIYDLIEL | 9 |
| B*2705 | 2BST | Influenza Nucleoprotein | SRYWAIRTR | 9 |
| B*2705 | 1HSA | Model Peptide Sequence | ARAAAAAAA | 9 |
| B*3501 | 1A9E | Peptide From Ebna3c-Protein From Ebv | LPPLDITPY | 9 |
| B*3501 | 2CIK | Epitope Derived From Cytochrome P450 | KPIVVLHGY | 9 |
| B*3501 | 2AXG | 10-mer peptide from BZLF1 trans-activator protein | APQPAPENAY | 10 |
| B*3508 | 2AXF | 10-mer peptide from BZLF1 trans-activator protein | APQPAPENAY | 10 |
| B*3508 | 3BWA | Hcmv 8-Mer Peptide From The Pp65 Protein Molecule | FPTKDVAL | 8 |

Fourty-nine HLA-restricted epitopes were used to identify the epitope conformational patterns restricted to human alleles A2*0201, B*0801, B*2705 and the supertype B*35.
