# Supplementary material for: Structural Allele-Specific Patterns Adopted by Epitopes in the MHC-I Cleft and Reconstruction of MHC:peptide Complexes to Cross-Reactivity Assessment
Source: PLoS One. 2010 Apr 26;5(4):e10353. doi: 10.1371/journal.pone.0010353 (PMC2860844; doi:10.1371/journal.pone.0010353)
Supplement: Table S2 — List of H-2Kb ligands available at PDB. (0.04 MB DOC) [file pone.0010353.s002.doc]

### Table S2 - List of H-2K^b^ ligands available at PDB.

| **PDB code** | **Epitope description** | **Sequence (aa)** | **N° of aa** |
| --- | --- | --- | --- |
| 1FO0*/2CLZ  1NAN | Naturally Processed Octapeptide Pbm1 | INFDFNTI | 8 |
| 1FZJ*/1KPU/  1NAM/2VAA/  1FZM/2MHA/1BQH | Vesicular Stomatitis Virus Nucleoprotein (NP_52-59_) | RGYVYQGL | 8 |
| 1KBG | Synthetic Glycopeptide Rgy8-6h-Gal2 | RGYVYMGL | 8 |
| 1FZK*/1FZO/  1KPV/2VAB | Sendai Virus Nucleoprotein (NP_324-332_) | FAPGNYPAL | 9 |
| 1G7P*/1VAD | Yeast Alpha-Glucosidase P1 (438-446) | SRDHSRTPM | 9 |
| 1G7Q* | Muc1 Vntr Peptide (180-187) | SAPDTRPA | 8 |
| 2FO4* | Peptide 8-mer derived from Mucin-1 | SAPDFRPL | 8 |
| 1KJ2/1KJ3* | Naturally Processed Octapeptide Pkb1 | KVITFIDL | 8 |
| 1LEG/1LEK*/ 1MWA/2CKB | Dev8_54-61_ (NADH-Ubiquinone Oxidoreductase MLRQ) | EQYKFYSV | 8 |
| 1LK2* | Insulin receptor (beta-subunit) derived Peptide (423-430) | GNYSFYAL | 8 |
| 1N59*/1S7Q | LCMV derived epitope (Gp33) | AVYNFATM | 8 |
| 1OSZ* | (L4v) Mutant Of Vesicular Stomatitis Virus Np | RGYLYQGL | 8 |
| 1P1Z*/1P4L/1VAC  3C8K/2QRI  2QRS/2QRT/3CVH | Ovalbumin derived Peptide  (257-264) | SIINFEKL | 8 |
| 1RJY*/1RKO/  1T0M/1T0N | Herpes Simplex Virus (HSV) Glycoprotein B derived Peptide | SSIEFARL | 8 |
| 1RJZ*/1RK1 | HSV Mutant S2E Glycoprotein B Peptide | SEIEFARL | 8 |
| 1S7R* | LCMV-Derived Gp33 Peptide - Escape Variants | KAVYNLATM | 9 |
| 1S7S* | LCMV-Derived Gp33 Peptide - Escape Variants | ALYNFATM | 8 |
| 1S7T* | LCMV-Derived Gp33 Peptide - Escape Variants | AVFNFATM | 8 |
| 2CLV*/2OL3 | Rbm5 derived Peptide (189-196) | SQYYYNSL | 8 |
| 1WBZ* | Influenza A peptide PB1_703-711_ | SSYRRPVGI | 9 |
| 1G6R | superagonist peptide SIYR | SIYRYYGL | 8 |
| 2ZSW* | 8-mer peptide from spike glycoprotein | R**A**YIFANI | 8 |
| 2ZSV* | 8-mer peptide from spike glycoprotein | R**A**QIFANI | 8 |

This table presents the 52 files containing the H-2K^b^ allele, available at PDB. Of these, 21 structures (*) presented epitopes with sequence differing in at least one amino acid. These 21 epitopes were used in the analyses. 1G6R presents an MHC interacting with the TCR. Since this interaction could affect the conformation of the epitope, this structure was excluded. 1KBG structure has a glicosilated residue and was also excluded.
