# Supplementary figures and images for: Structural Allele-Specific Patterns Adopted by Epitopes in the MHC-I Cleft and Reconstruction of MHC:peptide Complexes to Cross-Reactivity Assessment
Source: PLoS One. 2010 Apr 26;5(4):e10353. doi: 10.1371/journal.pone.0010353 (PMC2860844; doi:10.1371/journal.pone.0010353)

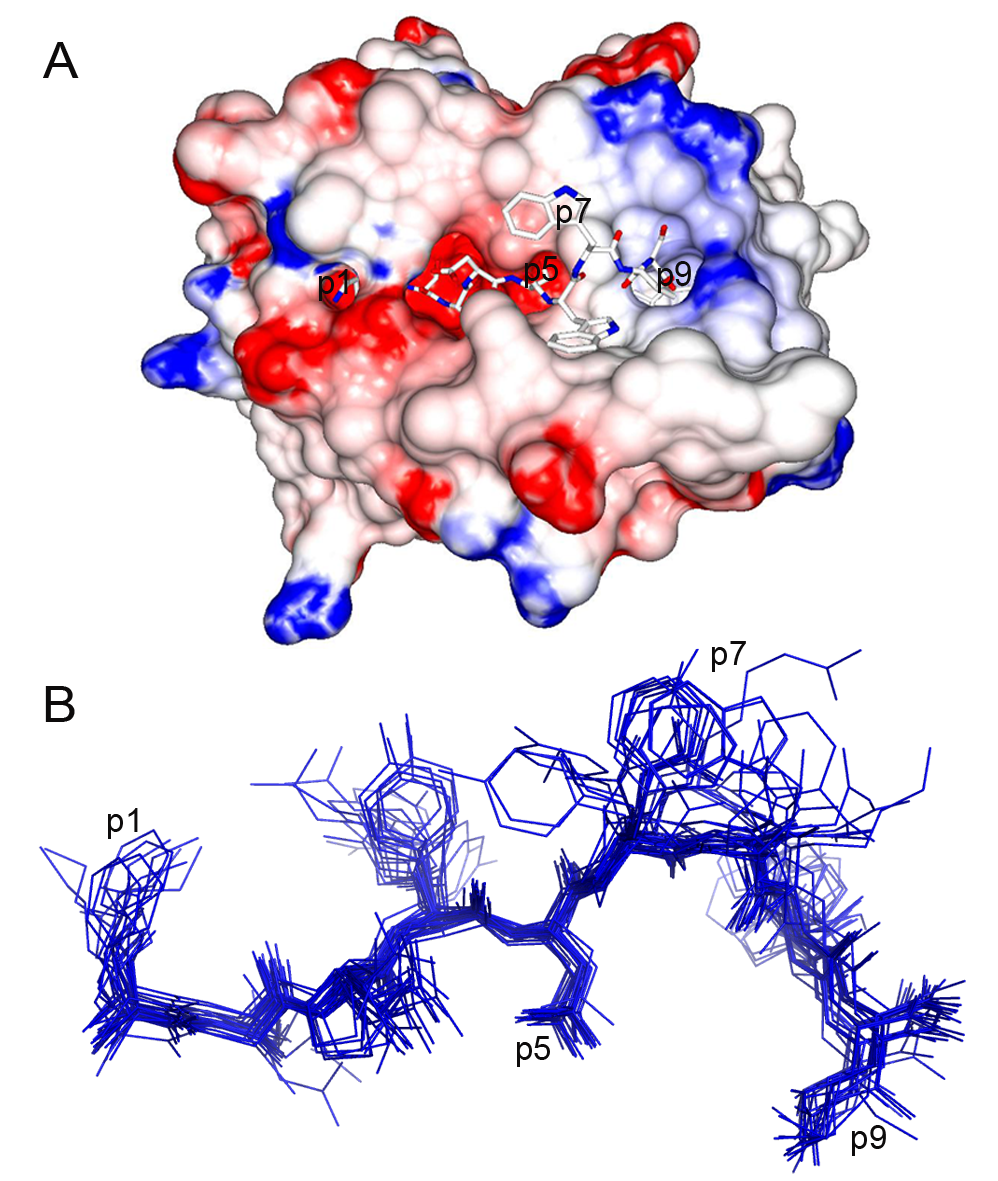

Supplement: Figure S1 — Structural organization of the H-2Db-restricted epitopes. A: Images of HBsAg30-39 epitope (presented in Ball and Stick_CPK) in the cleft of the H-2Db-allele (represented as surface, with negatively (red) and positively (blue) charged regions with a scale from −10 to +10 kiloteslas). Partial N-terminal ending of the epitope is hidden under MHC side chains (p2-3). Some regions of the peptides (p6-7) protract out to the MHC cleft. B. Superposition of 28 structures of H-2Db-restricted epitopes (Table 1), including side chains. It is possible to observe a higher variability in the protracted region as compared to the N-terminal and to the anchor sites (p5 and p9). The position of the side chains of amino acids 1, 5, 7 and 9 are shown in both images. (3.57 MB TIF) [file pone.0010353.s003.tif]

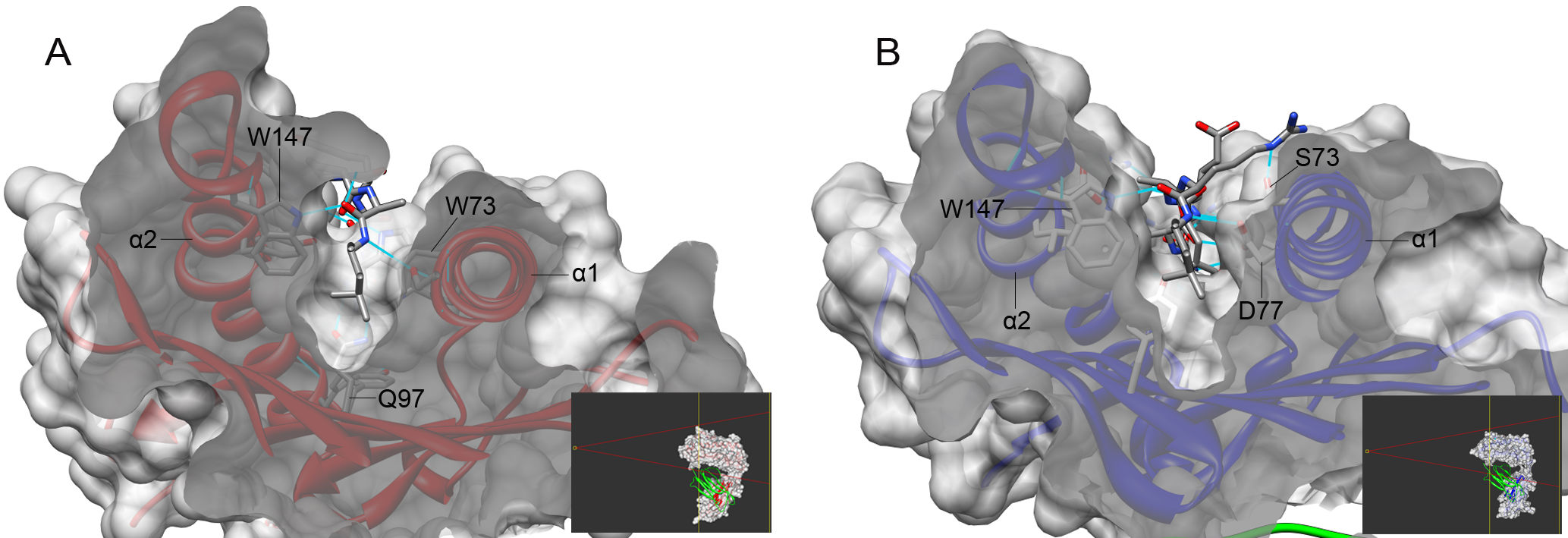

Supplement: Figure S2 — Topology of H2-Db and H2-Kb binding clefts. A: Crystal structure of an H-2Db allele (PDB access code 1CE6) is depicted as Ribbon and Surface. Epitopes inside the cleft are depicted as Sticks. Two tryptophanes of the MHC alpha-chain (W73 and W147) almost block the cleft, forcing the peptide to pass above them. B: Crystal structure of an H-2Kb allele (PDB access code 1RJY) is depicted with the same configuration. The absence of tryptophan (W73) results in a deeper cleft in this allele. (4.42 MB TIF) [file pone.0010353.s004.tif]

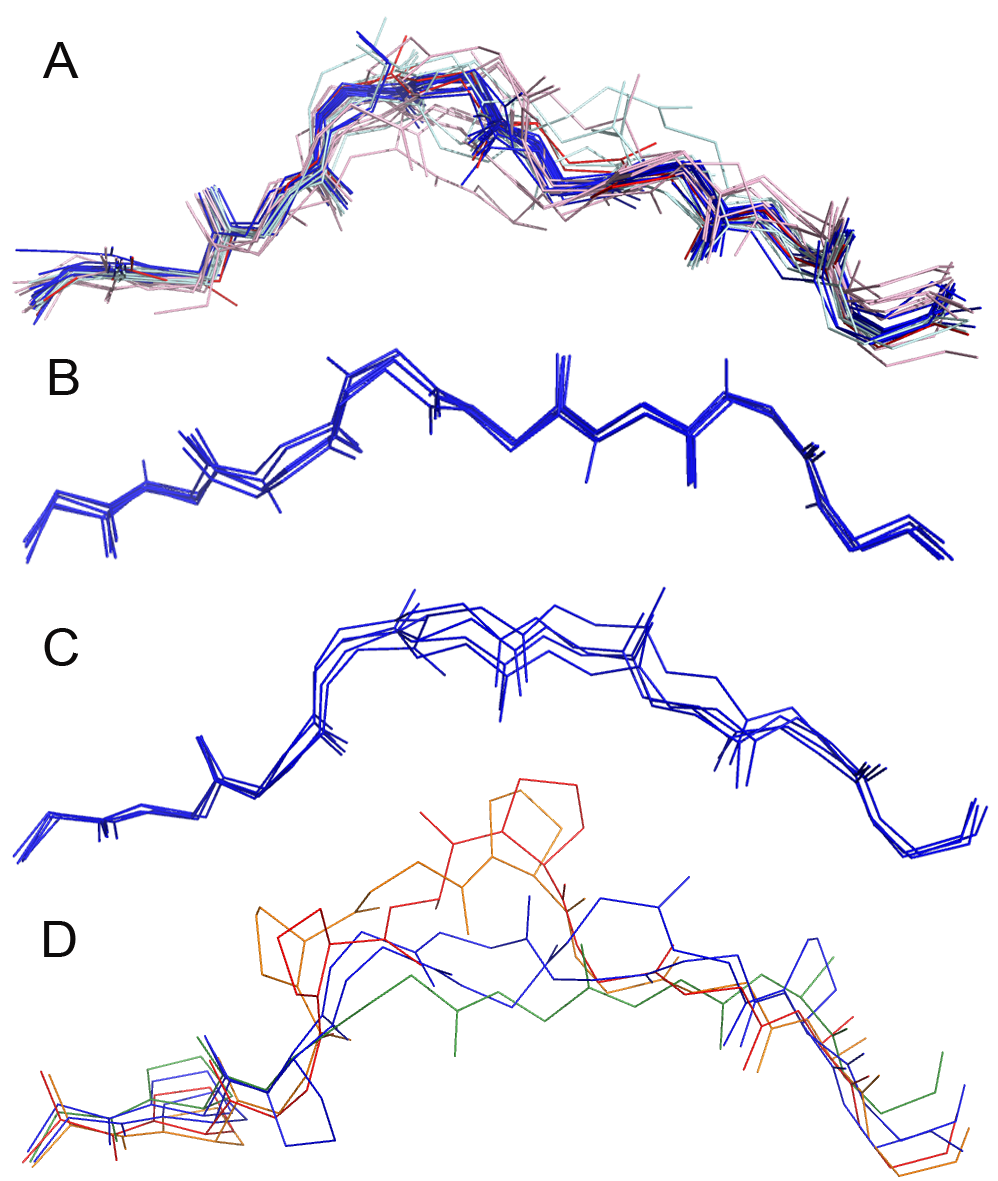

Supplement: Figure S3 — Conformational patterns of human MHC alleles. A: Thirty-four HLA-A*0201-restricted peptides (Table S1) were superposed using SPDBv program. Peptides sharing an A*0201-restricted pattern are depicted in blue. Exceptions to this pattern are depicted in cyan. Cancer-related peptides are depicted in light pink. Epitopes 1I7R and 1I7T are depicted in red (see Discussion). B: Superposition of five B*0801-restricted peptides. C: Superposition of five B*2705 restricted peptides. D: Superposition of three B*3501-restricted and two B*3508-restricted peptides. In this case, we have not enough structures to predict a pattern. Besides that, we can see that both B*3501-restricted 9-mers (blue) presented a similar conformation. In addition, a 10-mer peptide (APQPAPENAY) presented almost the same conformation when presented by B*3501 (orange) and B*3508 (red). A B*3508-restricted 8-mer is also depicted (green). (3.56 MB TIF) [file pone.0010353.s005.tif]

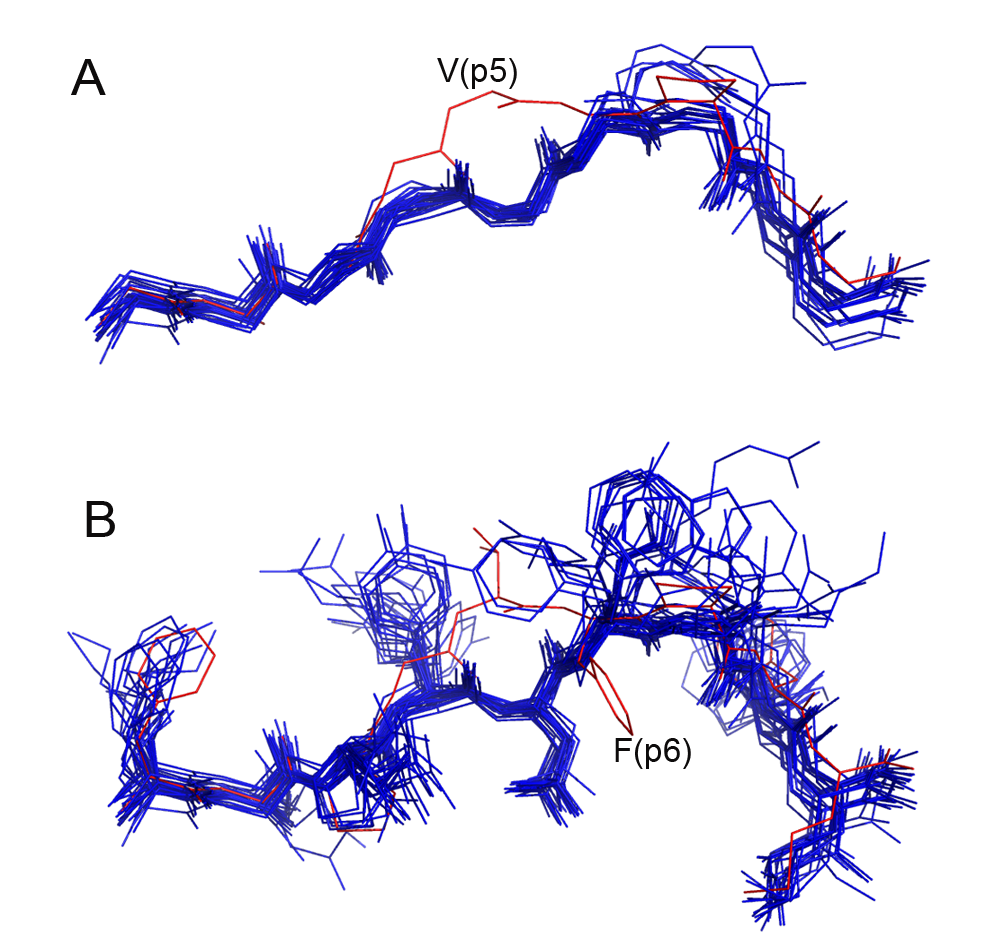

Supplement: Figure S4 — Exception to the H-2Db pattern. A: The 1BZ9 epitope (red) does not have the conventional amino acid in the anchor position (p5) and showed a significant deviation in the main chain when compared to other epitopes. B: The side chains of the epitope 1BZ9 (red) are in a similar conformation to other epitopes of this allele, except in a phenylalanine at position 6 of the epitope, which may be used as an alternative “anchor”. Observe the presence of an anchor amino acid in C-terminal and a hidden N-terminal extremity under the side chains of the MHC, characteristics that may contribute to the presentation of this unusual epitope. (2.86 MB TIF) [file pone.0010353.s006.tif]

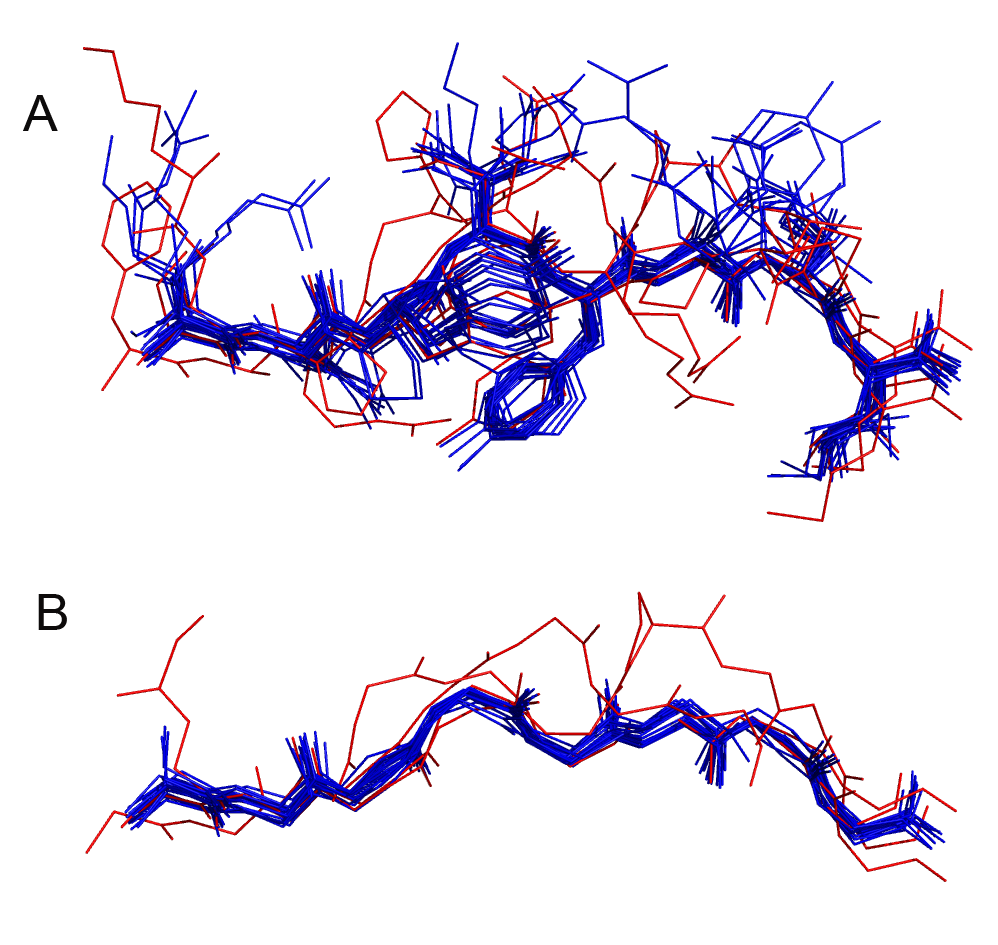

Supplement: Figure S5 — Structural pattern of H-2Kb restricted ligands. A: Twenty-two epitopes (see Table S2) restricted to this allele were superposed, four 9-mer (red) and 18 8-mer (blue). The H-2Kb restricted ligands, just like those restricted to the H-2Db allele, presented a higher identity in the side chains of the anchor positions than in the side chains oriented outside of the cleft. B: Backbone superposition of the 21 ligands shows a shared conformation among the epitopes with the same length (8-mer). The length adjustment, in this allele, seems to be in different positions, when compared to the H-2Db allele. (2.86 MB TIF) [file pone.0010353.s007.tif]

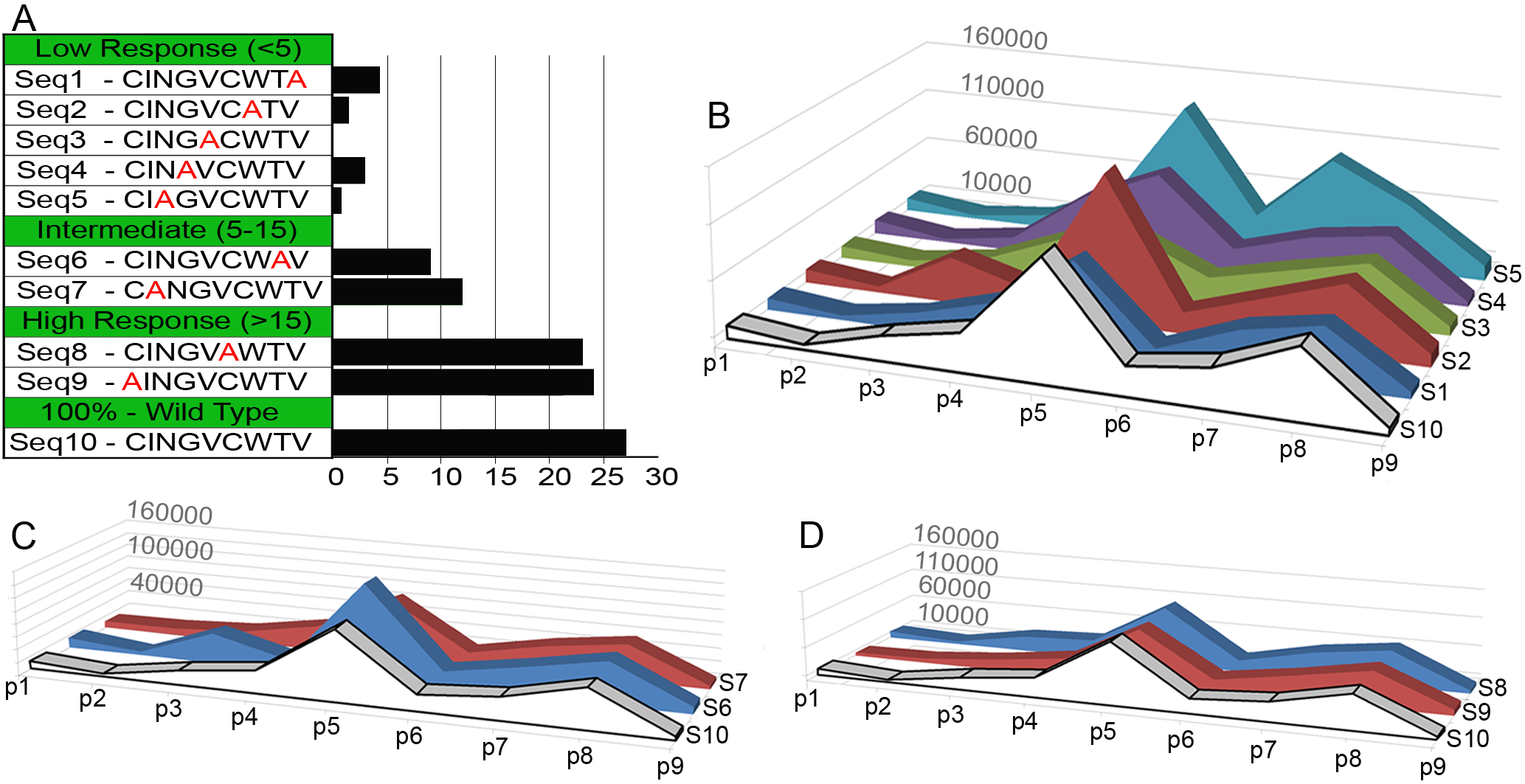

Supplement: Figure S6 — Analyze of HCV alanine exchanged peptides. The wild type HCV derived peptide (CINGVCWTV) and nine alanine exchanged peptides were analyzed. A: Sequences of 10 peptides are indicated. Level of IFN-Î3 production by CVNGVCWTV-specific CD8+ T-cells, induced by each sequence, are also represented [13]. Each bar (in black) represents the number of the Spot Forming Units (SFU/104 cells) produced by each of the peptides. Accessible Surface Area (ASA) plot of sequences that stimulates low (B), intermediated (C) and high (D) IFN-γ production are depicted. ASA values are measured in square angstroms. The wild type sequence (S10) was included in all plots. (6.62 MB TIF) [file pone.0010353.s008.tif]

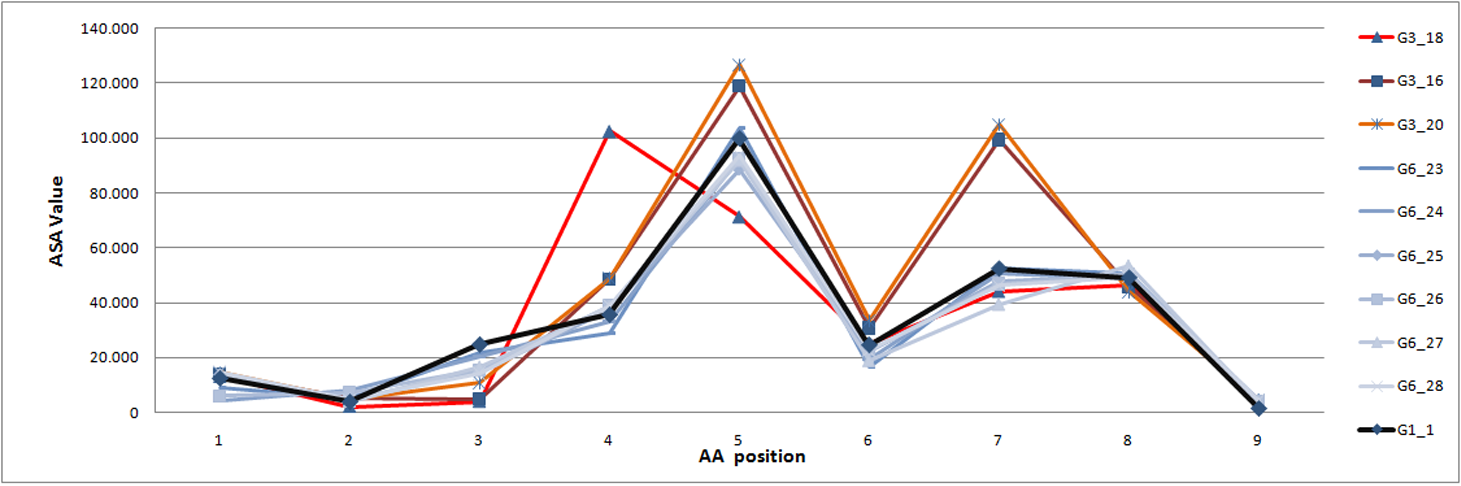

Supplement: Figure S7 — Flowchart of a new pMHC complex construction. ASA values of the wild type HCV derived peptide (CVNGVCWTV) and 28 naturally occurring NS31073-variants were analyzed. In agreement with experimental data, the ASA values from genotype 6 presented the same pattern of the wild type peptide and the peptides with higher deviation from this “ASA pattern” (G3_16, G3_18 and G3_20), presented the lowest levels of IFN-γ production in all ELISPOT assays (Fytili et al. 2008). (2.15 MB TIF) [file pone.0010353.s009.tif]

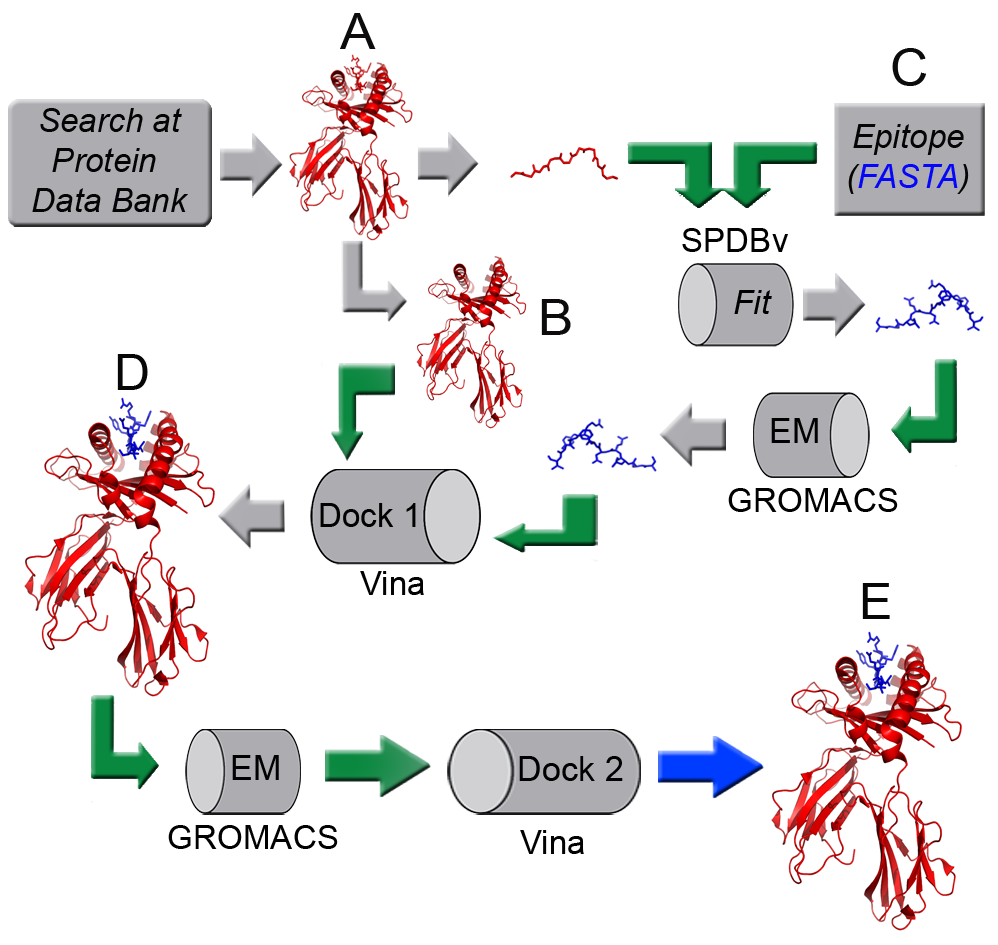

Supplement: Figure S8 — Flowchart of a new pMHC complex construction. Consider an epitope “C” whose structure in the context of a given MHC allele was not determined. A search at PDB is performed, looking for a PDB file containing the allele of interest presenting an epitope with the same length of the “c” epitope. In this example, we found the “A” complex. Using the SPDBV program, it was observed that the amino acid sequence of the epitope “c” is “Fit” on the 3D structure of the epitope present at the “A” complex. The generated structure of the epitope “c” is submitted to an energy minimization (EM), and is used as input for the docking with an “MHC donor” structure (B). In order to adjust the MHC to this new epitope, an EM of the complex “D” is performed. After minimization, epitope and MHC are separated and used as inputs to a second docking, which will generate the desired pMHC complex (E). For more information see methods. (2.86 MB TIF) [file pone.0010353.s010.tif]
